# Supplementary material for: Characterizing the services provided by family physicians in Ontario, Canada: A retrospective study using administrative billing data
Source: PLoS One. 2025 Jan 8;20(1):e0316554. doi: 10.1371/journal.pone.0316554 (PMC11709308; doi:10.1371/journal.pone.0316554)
Supplement: Attachment — Submitted filename: Response to Reviewers.docx [file pone.0316554.s001.docx]

Journal requirements:

When submitting your revision, we need you to address these additional requirements.

1. Please ensure that your manuscript meets PLOS ONE's style requirements, including those for file naming. The PLOS ONE style templates can be found at

<https://journals.plos.org/plosone/s/file?id=wjVg/PLOSOne_formatting_sample_main_body.pdf> and

<https://journals.plos.org/plosone/s/file?id=ba62/PLOSOne_formatting_sample_title_authors_affiliations.pdf>

I have reviewed both documents and updated the formatting as per the journal’s requirements.

2. Thank you for stating the following financial disclosure:

“RS is a co-investigator with DS as the principal investigator on a grant from the Northern Ontario Academic Medical Association (NOAMA) Clinical Innovation Opportunities Fund (C-18-19). Additional funding to SC from the Natural Sciences and Engineering Research Council (NSERC) of Canada supported this work.”

Please state what role the funders took in the study.  If the funders had no role, please state: "The funders had no role in study design, data collection and analysis, decision to publish, or preparation of the manuscript."

If this statement is not correct you must amend it as needed.

Please include this amended Role of Funder statement in your cover letter; we will change the online submission form on your behalf.

The financial disclosure has been updated to reflect that the funding agencies had no role in the study. As well, the text has been added to the cover letter for the revisions.

3. Thank you for stating the following in the Acknowledgments Section of your manuscript:

“This study contracted ICES Data & Analytic Services (DAS) and used de-identified data from the ICES Data Repository, which is managed by ICES with support from its funders and partners: Canada’s Strategy for Patient-Oriented Research (SPOR), the Ontario SPOR Support Unit, the Canadian Institutes of Health Research and the Government of Ontario. The opinions, results and conclusions reported are those of the authors. No endorsement by ICES or any of its funders or partners is intended or should be inferred.”

We note that you have provided funding information that is currently declared in your Funding Statement. However, funding information should not appear in the Acknowledgments section or other areas of your manuscript. We will only publish funding information present in the Funding Statement section of the online submission form.

Please remove any funding-related text from the manuscript and let us know how you would like to update your Funding Statement. Currently, your Funding Statement reads as follows:

“RS is a co-investigator with DS as the principal investigator on a grant from the Northern Ontario Academic Medical Association (NOAMA) Clinical Innovation Opportunities Fund (C-18-19). Additional funding to SC from the Natural Sciences and Engineering Research Council (NSERC) of Canada supported this work.”

Please include your amended statements within your cover letter; we will change the online submission form on your behalf.

The text above which is included in the Acknowledgements section does not reflect funding that we received for this study. It is an acknowledgement that is required by the data custodian (ICES – formerly known as the Institute for Clinical Evaluative Sciences) to reflect that their organization is supported by a number of other organizations. I found a previous ICES paper published in PLOS ONE that has a similar acknowledgement (<https://journals.plos.org/plosone/article?id=10.1371/journal.pone.0299005#ack>). The acknowledgements do vary depending on which administrative data sets are being accessed from each organization.

4. We note that you have indicated that there are restrictions to data sharing for this study. For studies involving human research participant data or other sensitive data, we encourage authors to share de-identified or anonymized data. However, when data cannot be publicly shared for ethical reasons, we allow authors to make their data sets available upon request. For information on unacceptable data access restrictions, please see <http://journals.plos.org/plosone/s/data-availability#loc-unacceptable-data-access-restrictions>.

Before we proceed with your manuscript, please address the following prompts:

a) If there are ethical or legal restrictions on sharing a de-identified data set, please explain them in detail (e.g., data contain potentially identifying or sensitive patient information, data are owned by a third-party organization, etc.) and who has imposed them (e.g., a Research Ethics Committee or Institutional Review Board, etc.). Please also provide contact information for a data access committee, ethics committee, or other institutional body to which data requests may be sent.

Part of the following text was included within the original Availability of data and materials section of the paper and has been updated to reflect that physician confidentiality and reidentification are a concern. The updated text reads: “***The dataset from this study is held securely in coded form at ICES. Due to concerns with physician confidentiality as a result of reidentification, the data cannot be shared. In addition, while data sharing agreements prohibit ICES from making the dataset publicly available, access may be granted to those who meet pre-specified criteria for confidential access, available at www.ices.on.ca/DAS. The full dataset creation plan and underlying analytic code are available from the authors upon request, understanding that the computer programs may rely upon coding templates or macros that are unique to ICES and are therefore either inaccessible or may require modification.”***

The text was updated to reflect that both physician confidentiality and data sharing agreements prohibit the data from being shared openly but that it can be accessed by contacting ICES.

b) If there are no restrictions, please upload the minimal anonymized data set necessary to replicate your study findings to a stable, public repository and provide us with the relevant URLs, DOIs, or accession numbers. Please see <http://www.bmj.com/content/340/bmj.c181.long> for guidelines on how to de-identify and prepare clinical data for publication. For a list of recommended repositories, please see <https://journals.plos.org/plosone/s/recommended-repositories>. You also have the option of uploading the data as Supporting Information files, but we would recommend depositing data directly to a data repository if possible.

Please update your Data Availability statement in the submission form accordingly.

Unfortunately, the data cannot be shared due to confidentiality concerns. Please see explanation above.

5. Your ethics statement should only appear in the Methods section of your manuscript. If your ethics statement is written in any section besides the Methods, please move it to the Methods section and delete it from any other section. Please ensure that your ethics statement is included in your manuscript, as the ethics statement entered into the online submission form will not be published alongside your manuscript.

The ethics statement and title were removed from the Supplementary Information section. A new section at the end of the Methods was added with a subheading title of Ethics approval and the following text: “***The Research Ethics Board at Lakehead University approved this study (#1466634).”***

6. Please include captions for your Supporting Information files at the end of your manuscript, and update any in-text citations to match accordingly. Please see our Supporting Information guidelines for more information: <http://journals.plos.org/plosone/s/supporting-information>.

I have added a section title for Supporting Information after the References and corrected the text reference on page 8.

7. Please review your reference list to ensure that it is complete and correct. If you have cited papers that have been retracted, please include the rationale for doing so in the manuscript text, or remove these references and replace them with relevant current references. Any changes to the reference list should be mentioned in the rebuttal letter that accompanies your revised manuscript. If you need to cite a retracted article, indicate the article’s retracted status in the References list and also include a citation and full reference for the retraction notice.

Within the revision we have added text with the appropriate citations and references added to the Reference section of the paper.

Reviewer #1: This is an excellent study: well planned, well analysed and well presented. It is of great importance for planning the education of family physicians and explaining to planners the exact situation now and in the future. My comments are only to improve the impact of their study.

Introduction

“Moreover, strong primary care systems have been shown to improve overall population health and reduce health system costs (2).”

[This is crucial to the importance and uptake of your research. Can you please provide perhaps from systematic reviews a summary that will convince readers]

The introduction paragraph was further expanded to describe why primary care is important to public health including its role in prevention, chronic disease management, reduction in health systems costs and improved health equity for under-served populations.

***“Moreover, strong primary care systems have been shown to improve overall population health through health prevention, management of chronic disease, improved patient outcomes and a reduction in health system costs [2]. Primary care is essential to public health since it addresses health inequities faced by underserved populations and by coordinating care across different providers [3].”***

“Although 69 previous studies have provided evidence for the varying scope of practice offered by different 70 family physicians in Canada, these mostly survey-based studies have suffered from low 71 response rates and recall bias when estimating the number and type of services being provided 72 (4”

[Can you provide examples of low response rates for important topics. This will greatly increase the importance of your study]

I have added a sentence that shows how response rates to the Canadian national physician workforce surveys have declined since 2001. The following sentence was added: “***For example, the response rate from a Canadian physician workforce survey in 2001 was approximately 50% [4], subsequent surveys in 2004 and 2007 had response rates of 30-35% [8] and finally a recent Canadian Medical Association workforce survey in 2017 and 2019 had a response rate of 12% [9,10].”***

“In addition, a much smaller proportion of specialists practice in rural 79 communities as compared to urban centres. Mian et al. (2017) described the unique challenges 80 in recruiting physicians to rural communities in northern Ontario (9). For rural populations, the 81 lack of access to primary care can lead to higher levels of chronic disease, lower life expectancy, 82 and overall poorer health status (10–12).”

[This is the essence of the problem. Can you provide some supporting stats please?]

The following sentence was added to quantify the discrepancy in health outcomes for those living in rural communities. “***For example, in rural areas the mortality due to respiratory and circulatory disease for both sexes was 10% higher, women had a 16% higher chance of mortality from diabetes and the life expectancy of males in rural areas was 2.8 years less than urban males [14]*.”**

Data sources

“We retrieved Ontario’s 2017 administrative healthcare data”

[readers will wonder why 2017 and not 2023. Please explain]

The analysis was started prior to the pandemic and there is typically a lag between when the data is generated and available on the ICES system. There are several sentences in the limitations section that point out that the workforce has likely change but that the methods and results will be important for future health human resource planning. The sentences below provide this context and have been revised.

***“Although the number of physicians practicing in Ontario has changed in the intervening years due to retirement and migration, we believe our analysis provides a thorough description of the family physician scope of practice in Ontario. This study also provides a methodology for future studies either in Ontario or other healthcare jurisdictions where health human resource planning is a challenge.”***

Typos
107 data was anonymized, [please change to were. Latin datum = singular, data = plural]

The text was corrected

139 in additional [change to in addition to]

The text was corrected.

193 provided a fewer number of [change to smaller]

The text was corrected.

204 Figure 2, however, the among different year in 205 practice in relatively equal [please rewrite]

This sentence was awkward and didn’t convey the original observation very well. It has been rewritten and should now convey the intended observation. *“****Notably, less than 5% of physicians practiced all six services shown in Fig 2 and they are distributed relatively equal amongst the range of years in practice.’***

I have published a study of readmission rates and mortality of seniors admitted to the Calgary hospitals 2013-2023. I sent it to the Premier, Health Minister and all the physician organisations. I was advised that the bureaucrats are the key persons who might take action on it.

At NOSM University we have an Associate Dean of Health Workforce Strategy. I collaborate with Dr. Newbery on a few different projects, although she is not a co-author on this work, she will be able to present the results to government officials that she meets with frequently.

Reviewer #2:

Well perfomed analysis of your local situation of services by family physicians. Unfortunately, it is very difficult to understand details for persons who are not familiar with your billing system. What do all these codes mean?

The following text was edited and added to the Methods to improve the description of the billing codes used by family physicians in Ontario. “***The OHIP billing database contains billing codes submitted to the provincial government by physicians to receive remuneration for the services provided to each patient. For example, when a physician assesses a patient within their clinic, they may bill the government using the A007 code while a physician in the emergency department may use the H133 code.”*** A list of all the billing codes used in the analysis can be found in the Supplementary Table 1.

Why is there such a low number of items for chonic care?

I assume that this is a reference to chronic disease management. Chronic disease management was not defined as a distinct service provided by the family physicians within our cohort. Although there are some billing codes related to diabetes management within the clinic, there is no comprehensive way to track all of the chronic diseases that a family physician would manage. It was assumed that chronic disease management would be part of a normal clinic service that family physician would provide.

What is about cooperations within one practice setting? Are they complementary?

If two or more physicians are practicing together and sharing a practice, their billings will be tracked within the OHIP billing code database as individual physicians. If the practice has a nurse practitioner, they are paid through a different process and the data set will not capture this cooperation. Across Ontario, the inability to identify where nurse practitioners are working and who they are seeing has been identified as a significant challenge for health human resource planning but the Ontario Ministry of Long-term Care has not made this data available yet.

Why is the number of young practicing doctors higher in rural regions? Are many of them moving to urban areas after some years?

This is an interesting question and one that we have noticed in the data. Unfortunately, the administrative data can answer many of the of 5 “W”s including who, what, where, and when but not the why. A longitudinal study could address where these physicians are moving within the province of Ontario. We have added the sentences below to the discussion. The additional text acknowledges this trend and references a qualitative study that examined physician retention in rural Ontario communities.

“***The results show that a higher proportion of early career physicians practice in rural communities. Although the administrative data provides little insight into why this is occurring, Jolicouer et al. (2022) found that physician retention in rural communities can be influenced by spousal employment opportunities, difficulty integrating into the community, physician burnout, and lack of resources.”***

If you wsh to publish your data in an international data you should give much more informations in the appendix and you should draw international comparisons

The following paragraph was added to the discussion under the subsection heading of “Family medicine practice internationally” to highlight the differences in practices across the world.

***“Western countries such as Canada and the United States have the most comprehensive scope of family medicine training, covering primary, emergency, hospital, long-term, maternal, and intrapartum care [31]. Australia’s rural practice stream matches this breadth, but its general practice stream is more limited, excluding emergency and intrapartum care. New Zealand, the United Kingdom, and Ireland offer narrower clinical training, with less focus on hospital and emergency care, though they still emphasize primary care and long-term care [31]. In terms of physician training time, Scandinavian countries have longer training programs, while Canada and Lebanon, have shorter two-year residencies [33]. The analysis from this study provides a comprehensive approach for policy and decision makers to quantify family physician scope internationally.”***
